# High expression of 5-hydroxymethylcytosine and isocitrate dehydrogenase 2 is associated with favorable prognosis after curative resection of hepatocellular carcinoma

**DOI:** 10.1186/1756-9966-33-32

**Published:** 2014-04-10

**Authors:** Wei-Ren Liu, Meng-Xin Tian, Lei Jin, Liu-Xiao Yang, Zhen-Bin Ding, Ying-Hao Shen, Yuan-Fei Peng, Jian Zhou, Shuang-Jian Qiu, Zhi Dai, Jia Fan, Ying-Hong Shi

**Affiliations:** 1 Department of Liver Surgery, Liver Cancer Institute, Zhongshan Hospital, Fudan University, 180 FengLin Road, Shanghai 200032, China; 2 Key Laboratory of Carcinogenesis and Cancer Invasion of Ministry of Education, Zhongshan Hospital, Fudan University, 180 FengLin Road, Shanghai, China; 3 Institutes of Biomedical Sciences, Fudan University, Shanghai, People’s Republic of China

**Keywords:** 5-hydroxymethylcytosine, Isocitrate dehydrogenase 2, Hepatocellular carcinoma, Immunohistochemistry, Prognosis

## Abstract

**Background:**

The expression of 5-hydroxymethylcytosine (5-hmC) and isocitrate dehydrogenase 2 (IDH2) is frequently downregulated in numerous cancers. 5-hmC and IDH2 expression in hepatocellular carcinoma (HCC) has yet to be determined.

**Methods:**

The immunohistochemical expression of 5-hmC and IDH2 were analyzed in tissue microarrays containing samples from 646 patients who had undergone hepatectomy for histologically proven HCC. The prognostic value of 5-hmC and IDH2 were evaluated by Cox regression and Kaplan-Meier analyses.

**Results:**

We discovered that low 5-hmC and IDH2 expression was associated with malignant behaviors. Low 5-hmC or IDH2 expression alone and combined 5-hmC and IDH2 expression were associated with lower overall survival (OS) rates and higher cumulative recurrence rates. Multivariate analysis indicated that 5-hmC or IDH2 and 5-hmC/IDH2 were independent prognostic indicators for OS and time to recurrence (TTR), which was confirmed in an independent validation cohort.

**Conclusions:**

5-hmC and IDH2 correlate with less aggressive tumor behavior in HCC. When 5-hmC and IDH2 are considered together, they serve as a prognostic marker in patients with surgically resected HCCs.

## Background

Liver cancer is the fifth most common cancer in men and the seventh most common in women, and hepatocellular carcinoma (HCC) is diagnosed in more than half a million of these liver cancer patients worldwide each year [1]. Despite intensive research, the prognosis of HCC remains poor, with an overall 5-year survival rate of approximately 26% in the United States [2]. There is a pressing need for novel biomarkers to identify the subset of patients with a high risk of recurrence and/or poor survival outcomes.

In the current cancer research landscape, epigenetics is a promising and expanding field [3-6]. DNA methylation, an important pattern of epigenetics, was historically believed to be a relatively stable chromatin modification, but the detection of the presence of 5-hmC facilitated a breakthrough in the field of epigenetic research [7,8]. 5-hmC, also known as the “sixth base”, was identified as an oxidant product of 5-methylcytosine (5mC) via the ten-eleven translocation (TET) family, which consists of TET1, -2, and -3. 5-hmC is abundant in embryonic stem (ES) cells and adult neural cells [8-10]. Currently, the biological prevalence of 5-hmC in cancer remains elusive. Lian et al. reported that the loss of 5-hmC was an epigenetic characteristic of melanoma with diagnostic and prognostic efficiency [11]. 5-hmC levels were high in low-grade tumors and decreased in malignant glioma [12]. Regarding gastroenteric tumors, 5-hmC was decreased in colorectal cancer (CRC) and gastric cancer [13]. In liver cancer, 5-hmC was also decreased compared with the surrounding normal tissue [14-16].

Isocitrate dehydrogenases (IDHs) catalyze the oxidative decarboxylation of isocitrate, which converts isocitrate to α-ketoglutarate (KG). The IDHs include IDH1 in the cytoplasm and IDH2 in the mitochondria, which catalyze an identical reaction [17] (Additional file 1: Figure S1). IDH1 and IDH2 mutations widely occur in gliomas and acute myeloid leukemia [18-21], leading to the production of 2-hydroxyglutarate (2-HG), which inhibits multiple α-KG-dependent dioxygenases, including the TET family of 5-mC hydroxylases (which results in decreased 5-hmC) [22]. Lian et al. found that IDH2 was significantly downregulated in melanoma [11]. However, 5-hmC and IDH2 expression in HCC have yet to be characterized in a large series of tumors with documented clinical, pathological, and molecular information.

In this study, we sought to determine the clinical relevance of 5-hmC and IDH2 protein expression in a large series of surgically resected HCCs using two cohorts. We studied the association between these two proteins and tumor history, as well as the patients’ clinical-pathologic features, including age, sex, stage, overall survival (OS), and time to recurrence (TTR). We found that combined 5-hmC and IDH2 protein expression was an independent prognostic factor for HCC patients after surgery.

## Materials and methods

### Patients and specimens

Archival specimens were obtained from two cohorts of consecutive patients with HCC who underwent curative resection at the Liver Cancer Institute, Zhongshan Hospital, Fudan University, between 2006 and 2007. The patient cohort inclusion and exclusion criteria included (a) accurate pathologic diagnosis of HCC, (b) complete clinicopathologic and follow-up data, (c) no anticancer treatment prior to curative liver resection, and (d) complete formalin-fixed, paraffin-embedded tissues. The histopathological diagnosis was determined according to the World Health Organization criteria. Tumor differentiation was graded using the Edmondson grading system [23]. Tumor staging was based on the 6th edition of the tumor-node-metastasis (TNM) classification of the International Union Against Cancer. Most patients (82.4%) had a hepatitis B virus background, and only two patients had hepatitis C virus. Almost all patients (316 of 318 for the training cohort and 325 of 328 for the validation cohort) were in the Child-Pugh A classification. The clinicopathologic characteristics of the two cohorts are summarized in Additional file 2: Table S1. Ethical approval was obtained from the Zhongshan Hospital Research Ethics Committee, and written informed consent was obtained from each patient.

### Follow-up and postoperative treatment

The follow-up data were summarized at the end of December 2011, with a median observation time of 52.2 months. The follow-up procedures were described in our previous study [23,24]. Postsurgical patient surveillance was undertaken as previously described [23,25]. OS was defined as the interval between the dates of surgery and death. TTR was defined as the interval between the dates of surgery and the dates of any diagnosed recurrence (intrahepatic recurrence and extrahepatic metastasis). For surviving patients, the data were censored at the date of death or last follow-up.

### Tissue microarray and immunohistochemistry

Tissue microarray (TMA) was conducted as previously described [26-28]. Briefly, all samples from the HCC patients were reviewed by three histopathologists and representative areas located away from necrotic and hemorrhagic materials were premarked in the paraffin blocks. Two core biopsies (1 mm in diameter) were taken from each representative tumor tissue and peritumoral tissue to construct the TMA slides. Consecutive sections measuring 4 μm were placed on 3-aminopropyltriethoxysilane-coated slides (Shanghai Biochip Co Ltd, Shanghai, People’s Republic of China).

Immunohistochemistry of the paraffin sections was performed using a two-step protocol (Novolink Polymer Detection System, Novocastra) according to the manufacturer’s instructions. Briefly, paraffin-embedded sections were deparaffinized and then rehydrated; after heat-induced antigen retrieval, endogenous peroxidases were blocked for 5 min using 0.3% H_2_O_2_, washed twice, and then incubated for 5 min in Serum-Free Protein Block (Novocastra), followed by incubation for 60 min in purified rabbit anti-human 5-hmC and rabbit anti-human IDH2 with DaVinci Green antibody diluent (Biocare Medical). The sections were incubated in a 3, 3-diaminobenzidine solution, counterstained with hematoxylin, dehydrated in ethanol, cleared in xylene, and coverslipped. Negative controls were treated in all assays (with the omission of primary antibodies). The sections were visualized using microscopic observation.

### Evaluation of the immunohistochemical findings

IHC staining was assessed by two independent pathologists without knowledge of the clinical and pathologic features of the cases. A negative control array was concurrently undertaken showing < 1% nuclear staining in all specimens. All specimens were evaluated according to the 0–4 grading criteria (based on the percentage of 5-hmC-positive cells) and 0–3 grading criteria (based on the staining intensity) [11]. The 5-hmC score was calculated as the score of the cell count × the score of intensity. The median 5-hmC score was used as a cut-off in subsequent analyses. For IDH2 quantification, photographs of three representative fields were captured under high-power magnification (200×) using Leica Qwin Plus v3 software; identical settings were used for each photograph. The 5-hmC and IDH2 density were counted using Image-Pro Plus v6.2 software (Media Cybernetics Inc., Bethesda, MD). The integrated optical density of the area positively stained for IDH2 in each photograph was calculated, and its ratio to the total area of each photograph was considered to be the IDH2 density. The median IDH2 density was used as a cut-off in subsequent analyses.

### Statistical analysis

The data were analyzed with SPSS 19.0 software, as previously described [23]. A *P* value <0.05 was considered statistically significant.

## Results

### Immunohistochemical features in TMA

**Figure 1:**
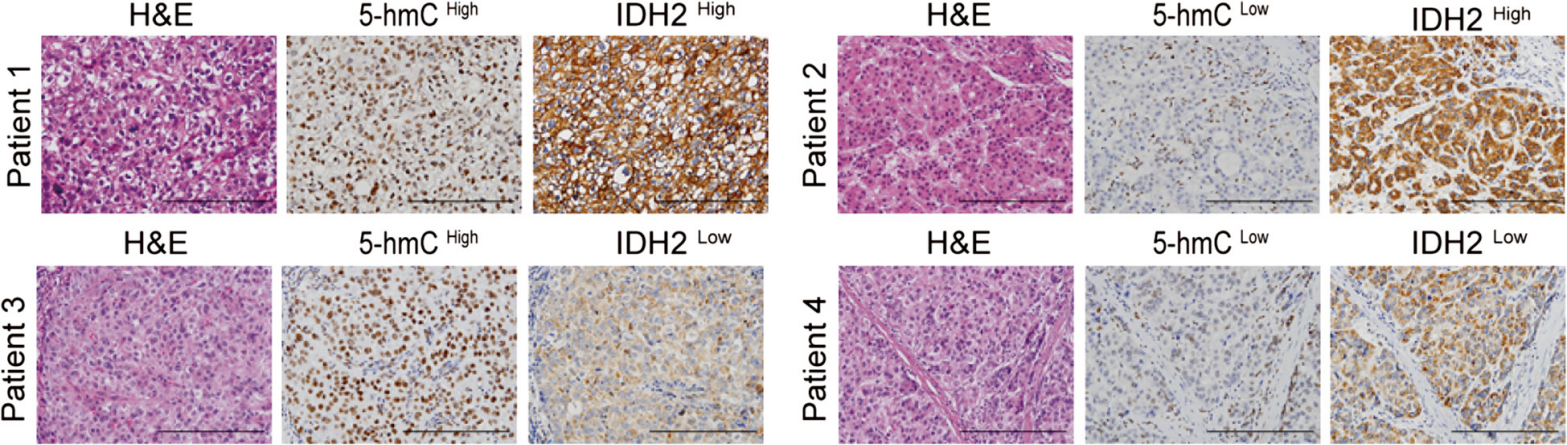
**Expression of 5-hmC and IDH2 in HCC samples (training cohort, n = 318).** Representative HCC tumor samples show the expression of 5-hmC (brown in the nucleus of HCC cells) and IDH2 (brown in the cytoplasm of HCC cells). Scale bar, 200×, 200 μm.

Using hematoxylin and eosin staining, the cancer cells were found to be relatively homogenous within a tumor (excluding necrotic, hemorrhagic, and fibrotic components). Representative cases of immunohistochemical staining are shown in Figure 1. We observed 5-hmC staining primarily on the nuclei of the tumor cells and hepatocytes; IDH2 staining was observed primarily in the cytoplasm of the HCC cells. Most of the stromal cells were negatively stained, although sporadic positive staining of these cells was observed.

### Correlations of 5-hmC and IDH2 expression with clinicopathologic characteristics

The correlations of 5-hmC and IDH2 expression with the clinicopathologic characteristics are shown in Table 1 and Additional file 2: Table S2. In the training cohort, 5-hmC expression correlated with sex (*P* =0.007) and AFP (*P* <0.001). IDH2 expression only correlated with tumor differentiation (*P* =0.017) (Table 1). In the validation cohort, 5-hmC expression correlated with sex (*P* =0.003), age (*P* =0.034), AFP (*P* <0.001), tumor number (*P* =0.02), and TNM stage (*P* =0.009). IDH2 expression correlated with HBsAg (*P* =0.015), AFP (*P* <0.001), and tumor differentiation (*P* =0.015) (Additional file 2: Table S2). Other clinical characteristics were not directly related to the expression of 5-hmC or IDH2.

**Table 1:** Summary of the correlations of 5-hmC and IDH2 protein expression with clinicopathological features in the training cohort (N = 318)

**Clinicopathological indexes**		**No. of patients**			**No. of patients**		
		**5-hmC **^**Low**^	**5-hmC **^**High**^	P^†^	**IDH2 **^**Low**^	**IDH2 **^**High**^	P^†^
Sex	Female	18	36	**0.007**	28	26	0.765
	Male	141	123		131	133	
Age(year)	≤50	55	65	0.247	60	60	1.000
	>50	104	94		99	99	
HBsAg	Negative	30	26	0.556	28	28	1.000
	Positive	129	133		131	131	
HCV	Negative	158	158	1.000	157	159	0.156
	Positive	1	1		2	0	
AFP	≤20	83	37	**<0.001**	58	62	0.644
	>20	76	122		101	97	
γ-GT(U/L)	≤54	87	81	0.500	78	90	0.178
	>54	72	78		81	69	
Liver cirrhosis	No	32	26	0.384	23	35	0.081
	Yes	127	133		136	124	
Tumor number	Single	131	134	0.652	134	131	0.652
	Multiple	28	25		25	28	
Tumor size(cm)	≤5	97	108	0.197	99	106	0.412
	>5	62	51		60	53	
Tumor encapsulation	Complete	94	88	0.496	93	89	0.650
	None	65	71		66	70	
Microvascular invasion	Absent	113	107	0.466	106	114	0.331
	Present	46	52		53	45	
Tumor differentiation	I + II	129	115	0.063	113	131	**0.017**
	III + IV	30	44		46	28	
TNM stage	I	98	93	0.567	93	98	0.567
	II + III	61	66		66	61	

*Abbreviations*: HBsAg, hepatitis B surface antigen; AFP, α-fetoprotein; γ-GT, γ-glutamyl transferase; TNM, tumor-node-metastasis.

^†^A *P*-value < 0.05 was considered statistically significant. *P*-values were calculated using the Pearson chi-square test. Boldface type indicates significant values.

### Association between combined 5-hmC and IDH2 expression and outcome in the training cohort

**Figure 2:**
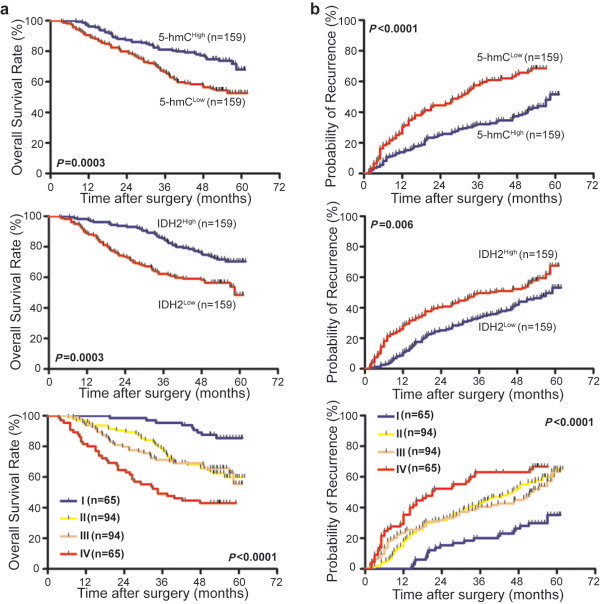
**5-hmC and IDH2 expression and prognostic value in HCC tissue (training cohort, N = 318).** Kaplan-Meier curves depiciting OS **(a)** and TTR **(b)** for 5-hmC expression, IDH2 expression, and combined 5-hmC/IDH2 expression. I, 5-hmC High/IDH2 High; II, 5-hmC Low/IDH2 High; III, 5-hmC High/IDH2 Low; IV, 5-hmC Low/IDH2 Low.

By the last follow-up in the training cohort (November 2011), 47.2% (150/318) of the patients had suffered a recurrence and 36.5% (116/318) had died. The 1-, 3-, and 5-year OS rates in the cohort were 83.6%, 67.6%, and 63.5% and the cumulative recurrence rates were 32.7%, 46.9%, and 52.8%, respectively. Additionally, we found that the 1-, 3-, and 5-year survival rates of the 5-hmC ^High^ patients were significantly higher than those of the 5-hmC ^Low^ group (87.4% vs. 79.9%, 77.4% vs. 57.9%, and 73.0% vs. 54.1%, respectively) (Figure 2a). Similarly, the 5-hmC ^Low^ patients had a poorer prognosis at 1, 3, and 5 years, with higher cumulative recurrence rates than the 5-hmC ^High^ patients (40.3% vs. 25.2%, 56.6% vs. 37.1%, and 61.6% vs. 44.0%, respectively) (Figure 2b). We also discovered that the 1-, 3-, and 5-year survival rates of the IDH2 ^High^ patients were significantly higher than those of the IDH2 ^Low^ group (93.7% vs. 73.6%, 76.7% vs. 58.5%, and 71.7% vs. 55.3%, respectively) (Figure 2a). Similarly, the IDH2 ^Low^ patients had a poorer prognosis at 1-, 3-, and 5- years, with higher cumulative recurrence rates than the IDH2 ^High^ patients (40.3% vs. 25.2%, 52.2% vs. 41.5%, and 58.5% vs. 47.2%, respectively) (Figure 2b). Furthermore, we evaluated the combined effect of 5-hmC and IDH2 expression. We found that the 1-, 3-, and 5-year OS rates in the 5-hmC ^Low^/IDH2 ^Low^ patients were 64.6%, 43.1%, and 43.1%, respectively, which were significantly lower than those in the 5-hmC ^High^/IDH2 ^High^ patients (98.5%, 89.2%, and 86.2%, respectively) (Figure 2a). The cumulative recurrence rates in the 5-hmC ^Low^/IDH2 ^Low^ patients were 52.3%, 63.1% and 66.2%, respectively, which were significantly higher than those in the 5-hmC ^High^/IDH2 ^High^ patients (15.4%, 26.2% and 30.8%, respectively) (Figure 2b).

Univariate analysis revealed that 5-hmC (*P* <0.001 and *P* = 0.001), IDH2 (*P* <0.001 and *P* = 0.006), and 5-hmC/IDH2 combined (*P* <0.001 and *P* <0.001) were associated with OS and TTR. γ-GT, tumor number, tumor size, microvascular invasion, and TNM stage were predictors of OS and TTR. Moreover, AFP was only associated with OS, and liver cirrhosis was only associated with TTR (Table 2).

**Table 2:** Summary of univariate and multivariate analyses of 5-hmC and IDH2 protein expression associated with survival and recurrence in the training cohort (N = 318)

**Factor**	**OS**				**TTR**			
	**Multivariate**				**Multivariate**			
	**Univariate P**	**Hazard ratio**	**95% CI**	P^†^	**Univariate P**	**Hazard ratio**	**95% CI**	P^†^
Sex (female vs. male)	0.959			NA	0.083			NA
Age, years (≤50 vs. >50)	0.772			NA	0.597			NA
HBsAg (negative vs. positive)	0.983			NA	0.491			NA
AFP, ng/ml (≤20 vs. >20)	**0.041**	1.893	1.257–2.852	**0.002**	0.230			NA
γ-GT, U/L (≤54 vs. >54)	**0.006**	1.619	1.118–2.343	**0.011**	**0.003**	1.547	1.138–2.102	**0.005**
Liver cirrhosis (no vs. yes)	0.077			NA	**0.009**	1.824	1.135–2.930	**0.013**
Tumor number (single vs. multiple)	**0.003**			NS	**0.002**	1.651	1.135–2.402	**0.009**
Tumor size, cm (≤5 vs. >5)	**0.009**			NS	**0.041**			NS
Tumor encapsulation (complete vs. none)	0.261			NA	0.166			NA
Microvascular invasion (no vs. yes)	**0.003**			NS	**0.001**	1.775	1.287–2.448	**<0.001**
Tumor differentiation (I-II vs. III-IV)	0.138			NA	0.053			NA
TNM stage (I vs. II III)	**<0.001**	2.048	1.412–2.971	**<0.001**	**<0.001**	1.649	1.134–2.397	**0.009**
5-hmC (low vs. high)	**<0.001**	0.316	0.211–0.472	**<0.001**	**0.001**	0.462	0.335–0.636	**<0.001**
IDH2 (low vs. high)	**<0.001**	0.405	0.275–0.594	**<0.001**	**0.006**	0.591	0.432–0.810	**0.001**
Combination of 5-hmC and IDH2	**<0.001**			**<0.001**	**<0.001**			**<0.001**
I versus II	**0.002**	3.987	1.890–8.413	**<0.001**	**0.001**	2.651	1.576–4.461	**<0.001**
I versus III	**0.002**	3.359	1.607–7.025	**0.001**	**0.003**	2.098	1.247–3.530	**0.005**
I versus IV	**<0.001**	8.908	4.215–18.825	**<0.001**	**<0.001**	3.891	2.270–6.671	**<0.001**

*Abbreviations*: OS, overall survival; TTR time to recurrence; AFP, α-fetoprotein; γ-GT, γ-glutamyl transferase; TNM, tumor-node-metastasis; CI, confidence interval; NA, not adopted; NS, not significant.

^†^Cox proportional hazards regression. Boldface type indicates significant values.

I, 5-hmC ^High^/IDH2 ^High^; II, 5-hmC ^Low^/IDH2 ^High^; III, 5-hmC ^High^/IDH2 ^Low^; IV, 5-hmC ^Low^/IDH2 ^Low^.

The individual clinicopathological features that presented significance in the univariate analysis were adopted as covariates in a multivariate Cox proportional hazards model for further analysis. 5-hmC and IDH2 were prognostic indicators of OS (*P* <0.001 and *P* <0.001) and TTR (*P* <0.001 and *P* =0.001). When 5-hmC was combined with IDH2, we found that 5-hmC/IDH2 was also an independent prognostic indicator of both OS (*P* <0.001) and TTR (*P* <0.001) (Figure 2 and Table 2).

### Validation analysis of the better outcome of patients in the validation cohort with 5-hmC ^High^/IDH2 ^High^ expression

**Figure 3:**
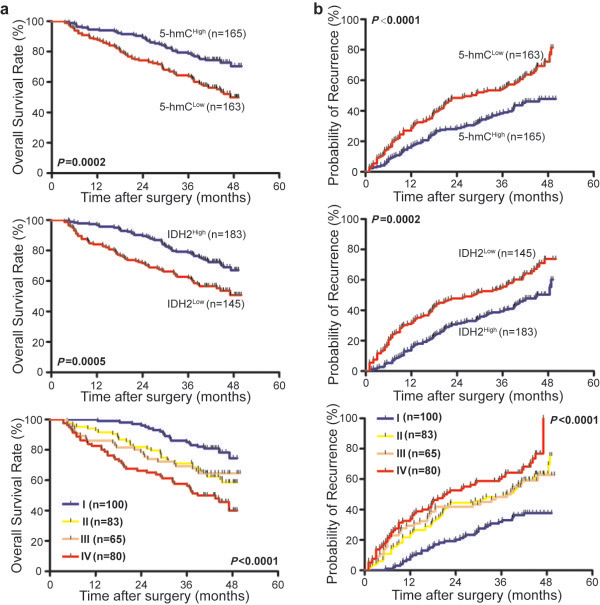
**5-hmC and IDH2 expression and prognostic value in HCC tissue (validation cohort, N = 328).** Kaplan-Meier curves depiciting OS **(a)** and TTR **(b)** for 5-hmC expression, IDH2 expression, and combined 5-hmC/IDH2 expression. I, 5-hmC ^High^/IDH2 ^High^; II, 5-hmC ^Low^/IDH2 ^High^; III, 5-hmC ^High^/IDH2 ^Low^; IV, 5-hmC ^Low^/IDH2 ^Low^.

To validate our findings of better outcomes in patients with 5-hmC ^High^/IDH2 ^High^ expression, we studied a validation cohort that included 328 surgically resected HCC tumors. Briefly, we found that the 1- and 3-year OS rates in the 5-hmC ^Low^/IDH2 ^Low^ patients were 66.3% and 46.3%, respectively, which were significantly lower than those in the 5-hmC ^High^/IDH2 ^High^ patients (97.0% and 79.0%, respectively) (Figure 3a). The cumulative recurrence rates in the 5-hmC ^Low^/IDH2 ^Low^ patients were 52.5% and 71.3%, respectively, which were significantly higher than those in the 5-hmC ^High^/IDH2 ^High^ patients (19.0% and 36.0%, respectively) (Figure 3b).

Univariate analysis revealed that 5-hmC (*P* <0.001 and *P* <0.001), IDH2 (*P* =0.001 and *P* <0.001), and 5-hmC/IDH2 combined (*P* <0.001 and *P* <0.001) were associated with OS and TTR. In a multivariate Cox proportional hazards model, 5-hmC and IDH2 were prognostic indicators of OS (*P* =0.005 and *P* =0.005) and TTR (*P* =0.008 and *P* =0.02). When 5-hmC and IDH2 were combined, we found that 5-hmC/IDH2 was also an independent prognostic indicator of both OS (*P* =0.007) and TTR (*P* =0.009) (Additional file 2: Table S3).

## Discussion

To date, the available data on 5-hmC and IDH2 in HCC have been limited. In this study, we investigated the clinical relevance of 5-hmC and IDH2 protein expression in two large cohorts (n = 646) of surgically resected HCCs with 318 cases and 328 cases, respectively.

We determined that high 5-hmC expression was significantly associated with favorable features in HCC patients. This finding may be substantiated by the fact that aggressive histopathological characteristics, including a high AFP level was significantly more frequent in patients with low 5-hmC expression than in those with high expression in training cohort. And a high AFP level, more tumor number, and an advanced TNM staging of HCC were more detected in those patients with low 5-hmC expression in validation cohort. This indicated that 5-hmC may be a powerful prognostic indicator in HCC. 5-hmC, an oxidation product of 5mC via the TET family (which consists of TET1, -2, and -3), is abundant in ES cells and adult neural cells [8]. The relationship between 5-hmC and tumors is emerging through a number of studies [8,11,29]. In liver cancer research, 5-hmC expression was decreased in liver cancer compared with the surrounding normal tissue [14,15]. Although previous studies have addressed 5-hmC protein expression using IHC in archived HCC tissues, the number of cases is limited and lacks further validation. Our study represents the largest analysis of 5-hmC protein expression in HCC.

We also detected significant correlations between low IDH2 expression and HBsAg background, a high level of AFP, and low-grade tumor differentiation. IDH2, an IDH (which convert isocitrate to α-KG), is frequently mutated in cancer, particularly in secondary glioblastoma [30], cytogenetically normal acute myeloid leukemia (AML) [31], cartilaginous tumors [32], and intrahepatic cholangiocarcinoma [33]. The pathophysiological function of the *R*-enantiomer of 2-hydroxylglutarate (*R*-2-HG) is the driving force of IDH1/2 mutation-induced tumorigenesis [22]. In melanoma, IDH2 is frequently downregulated, and the overexpression of IDH2 in a zebrafish melanoma model has been shown to increase the level of 5-hmC, resulting in prolonged tumor-free survival [11]. In our group, the preliminary experimental results indicated a tumor suppressor role for IDH2 in HCC (unpublished data); however, the expression of mutated IDH2, the mechanisms of IDH2 mutation, and the precise role of IDH2 in HCC remain under investigation.

One of most notable findings of our study was that the expression of 5-hmC or IDH2 alone, as well as the expression of the combination of 5-hmC and IDH2, was significantly correlated with OS and TTR in two cohorts. Thus, we made a direct comparison of prognosis between four subgroups (5-hmC ^High^/IDH2 ^High^, 5-hmC ^Low^/IDH2 ^High^, 5-hmC ^High^/IDH2 ^Low^, and 5-hmC ^Low^/IDH2 ^Low^) in the training cohort. As expected, patients with 5-hmC ^High^/IDH2 ^High^ expression had a significantly better OS and TTR than the patients in the other 3 groups in both univariate and multivariate analyses. These interesting observations were confirmed in a second cohort (validation cohort) that exhibited clinical-pathological features similar to the first cohort (training cohort).

In addition to genetic alterations, epigenetic alterations were also considered to participate in carcinogenesis [34]. It is also plausible that the two mechanisms can coexist and interact, giving birth to the observed hot-spot tumor heterogeneity [35,36]. The mechanisms of this interaction are currently the chief investigational pursuit of our laboratory. In this study, we determined the prognostic value of 5-hmC and IDH2 in HCC; further investigations are in progress.

The major limitation of the present work was its retrospective nature. Moreover, it is noteworthy that most HCC patients in China have a hepatitis B virus-positive background, which differs from studies in Japan, Europe, and the United States.

To the best of our knowledge, this is the first paper demonstrating the implications of 5-hmC and IDH2 in HCC. Our findings indicate that a high expression of 5-hmC and IDH2 predicts comparably less aggressive tumor behavior. Importantly, 5-hmC expression (particularly when combined with IDH2 expression) enables us to more accurately predict the true prognosis of HCC patients. Moreover, given the proposed epigenetic nature of 5-hmC and IDH2, the therapeutic manipulation of 5-hmC and IDH2 will assist in guiding clinical strategies.

## Conclusions

In summary, 5-hmC and IDH2 correlate with less aggressive tumor behavior in HCC. Low 5-hmC or IDH2 expression alone and combined 5-hmC and IDH2 expression were associated with lower OS rates and higher cumulative recurrence rates. When 5-hmC and IDH2 are considered together, they serve as a prognostic marker in patients with surgically resected HCCs.

## Abbreviations

5-hmC: 5-hydroxymethylcytosine; IDH2: Isocitrate dehydrogenase 2; HCC: Hepatocellular carcinoma; OS: Overall survival; TTR: Time to recurrence; TMA: Tissue microarray.

## Competing interests

The authors declare no competing interests.

## Authors’ contributions

WRL and MXT contributed equally to this work. All authors read and approved the final manuscript.

## Supplementary Material

Additional file 1: Figure S1 Diagram figure to summarize the biological functions of IDH2 and 5-hmC. Click here for file

Additional file 2: Table S1 Summary of the clinicopathological features of the training and validation cohort. **Table S2.** Summary of the correlations of 5-hmC and IDH2 protein expression with clinicopathological features in validation cohort (N=328). **Table S3.** Summary of univariate and multivariate analyses of 5-hmC and IDH2 protein expression associated with survival and recurrence in validation cohort (N=328). Click here for file
