# Supplementary material for: High expression of 5-hydroxymethylcytosine and isocitrate dehydrogenase 2 is associated with favorable prognosis after curative resection of hepatocellular carcinoma
Source: J Exp Clin Cancer Res. 2014 Apr 10;33(1):32. doi: 10.1186/1756-9966-33-32 (PMC4081660; doi:10.1186/1756-9966-33-32)

**Additional file 1. Supplementary Figure Legend**

Figure S1. Diagram figure to summarize the biological functions of IDH2 and 5-hmC.


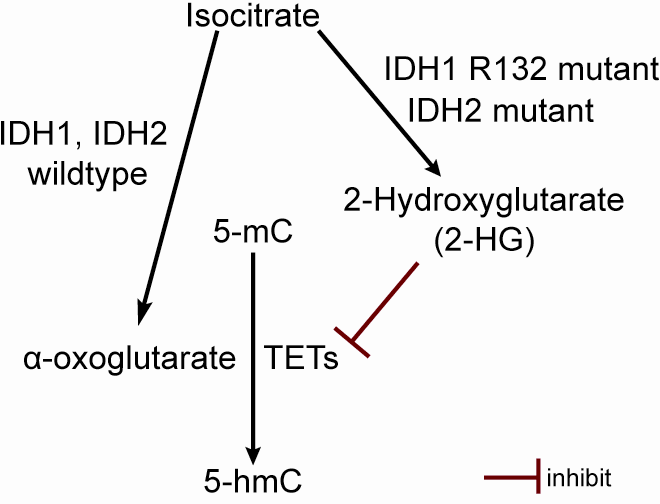

Supplement: Additional file 1: Figure S1 — Diagram figure to summarize the biological functions of IDH2 and 5-hmC. [file 1756-9966-33-32-S1.doc]
