# Supplementary material for: High expression of 5-hydroxymethylcytosine and isocitrate dehydrogenase 2 is associated with favorable prognosis after curative resection of hepatocellular carcinoma
Source: J Exp Clin Cancer Res. 2014 Apr 10;33(1):32. doi: 10.1186/1756-9966-33-32 (PMC4081660; doi:10.1186/1756-9966-33-32)
Supplement: Additional file 2: Table S1 — Summary of the clinicopathological features of the training and validation cohort. Table S2. Summary of the correlations of 5-hmC and IDH2 protein expression with clinicopathological features in validation cohort (N=328). Table S3. Summary of univariate and multivariate analyses of 5-hmC and IDH2 protein expression associated with survival and recurrence in validation cohort (N=328). [file 1756-9966-33-32-S2.docx]

**Additional file 2: Supplementary Tables**

**Supplementary Table S1. Summary of the clinicopathological features of the training and validation cohort**

| Clinicopathological indexes |  | Training Cohort (N=318) | | Validation Cohort(N=328) | |
| --- | --- | --- | --- | --- | --- |
|  |  | N | % | N | % |
| Sex | Female | 54 | 17.0 | 42 | 12.8 |
|  | Male | 264 | 83.0 | 286 | 87.2 |
| Age(year) | ≤50 | 120 | 37.7 | 132 | 40.2 |
|  | >50 | 198 | 62.3 | 196 | 59.8 |
| HBsAg | Negative | 56 | 17.6 | 65 | 19.8 |
|  | Positive | 262 | 82.4 | 263 | 80.2 |
| HCV | Negative | 316 | 99.4 | 323 | 98.5 |
|  | Positive | 2 | .6 | 5 | 1.5 |
| AFP | ≤20 | 120 | 37.7 | 132 | 40.2 |
|  | >20 | 198 | 62.3 | 196 | 59.8 |
| γ-GT(U/L) | ≤54 | 168 | 52.8 | 148 | 45.1 |
|  | >54 | 150 | 47.2 | 180 | 54.9 |
| Liver cirrhosis | No | 58 | 18.2 | 52 | 15.9 |
|  | Yes | 260 | 81.8 | 276 | 84.1 |
| Tumor number | Single | 265 | 83.3 | 277 | 84.5 |
|  | Multiple | 53 | 16.7 | 51 | 15.5 |
| Tumor size(cm) | ≤5 | 205 | 64.5 | 218 | 66.5 |
|  | >5 | 113 | 35.5 | 110 | 33.5 |
| Tumor encapsulation | complete | 182 | 57.2 | 160 | 48.8 |
|  | none | 136 | 42.8 | 168 | 51.2 |
| Microvascular invasion | absence | 220 | 69.2 | 238 | 72.6 |
|  | present | 98 | 30.8 | 90 | 27.4 |
| Tumor differentiation | I+II | 244 | 76.7 | 237 | 72.3 |
|  | III+IV | 74 | 23.3 | 91 | 27.7 |
| TNM stage | I | 191 | 60.1 | 208 | 63.4 |
|  | II+III | 127 | 39.9 | 120 | 36.6 |

Abbreviations: HBsAg, hepatitis B surface antigen; AFP, α-fetoprotein; γ-GT, γ-glutamyl transferase; TNM, tumor-nodes-metastasis.

**Supplementary Table S2. Summary of the correlations of 5-hmC and IDH2 protein expression with clinicopathological features in validation cohort (N=328)**

| Clinicopathological indexes |  | No. of patients | | | No. of patients | | |
| --- | --- | --- | --- | --- | --- | --- | --- |
|  |  | 5-hmC ^Low^ | 5-hmC ^High^ | P† | IDH2 ^Low^ | IDH2 ^High^ | P† |
| Sex | Female | 30 | 12 | **0.003** | 21 | 21 | 0.418 |
|  | Male | 133 | 153 |  | 124 | 162 |  |
| Age(year) | ≤50 | 75 | 57 | **0.034** | 55 | 77 | 0.447 |
|  | >50 | 88 | 108 |  | 90 | 106 |  |
| HBsAg | Negative | 31 | 34 | 0.718 | 20 | 45 | **0.015** |
|  | Positive | 132 | 131 |  | 125 | 138 |  |
| HCV | Negative | 160 | 163 | 0.642 | 144 | 179 | 0.272 |
|  | Positive | 3 | 2 |  | 1 | 4 |  |
| AFP | ≤20 | 44 | 88 | **<0.001** | 41 | 91 | **<0.001** |
|  | >20 | 119 | 77 |  | 104 | 92 |  |
| γ-GT(U/L) | ≤54 | 71 | 77 | 0.572 | 58 | 90 | 0.097 |
|  | >54 | 92 | 88 |  | 87 | 93 |  |
| Liver cirrhosis | No | 23 | 29 | 0.390 | 18 | 34 | 0.129 |
|  | Yes | 140 | 136 |  | 127 | 149 |  |
| Tumor number | Single | 130 | 147 | **0.020** | 121 | 156 | 0.655 |
|  | Multiple | 33 | 18 |  | 24 | 27 |  |
| Tumor size(cm) | ≤5 | 105 | 113 | 0.435 | 89 | 129 | 0.083 |
|  | >5 | 58 | 52 |  | 56 | 54 |  |
| Tumor encapsulation | complete | 80 | 80 | 0.914 | 68 | 92 | 0.543 |
|  | none | 83 | 85 |  | 77 | 91 |  |
| Microvascular invasion | absence | 112 | 126 | 0.120 | 100 | 138 | 0.194 |
|  | present | 51 | 39 |  | 45 | 45 |  |
| Tumor differentiation | I+II | 113 | 124 | 0.239 | 95 | 142 | **0.015** |
|  | III+IV | 50 | 41 |  | 50 | 41 |  |
| TNM stage | I | 92 | 116 | **0.009** | 89 | 119 | 0.496 |
|  | II+III | 71 | 49 |  | 56 | 64 |  |

Abbreviations: HBsAg, hepatitis B surface antigen; AFP, α-fetoprotein; γ-GT, γ-glutamyl transferase; TNM, tumor-nodes-metastasis.

† A *P*-value < 0.05 was considered statistically significant. *P*-values were calculated using the Pearson chi-square test. Boldface type indicates significant values.

**Supplementary Table S3. Summary of univariate and multivariate analyses of 5-hmC and IDH2 protein expression associated with survival and recurrence in validation cohort (N=328)**

| Factor | OS | | | | TTR | | | |
| --- | --- | --- | --- | --- | --- | --- | --- | --- |
|  | Multivariate | | | | Multivariate | | | |
|  | Univariate P | Hazard  Ratio | 95% CI | P† | Univariate P | Hazard  Ratio | 95% CI | P† |
| Sex (female vs. male) | 0.783 |  |  | NA | 0.510 |  |  | NA |
| Age, years (≤50 vs. >50) | 0.937 |  |  | NA | 0.454 |  |  | NA |
| HBsAg (negative vs. positive) | 0.141 |  |  | NA | **0.002** | 2.073 | 1.342-3.203 | **0.001** |
| AFP, ng/ml (≤20 vs. >20) | **0.010** |  |  | NS | **<0.001** | 1.568 | 1.120-2.196 | **0.009** |
| γ-GT, U/L (≤54 vs. >54) | **0.006** |  |  | NS | **0.045** |  |  | NS |
| Liver cirrhosis (no vs. yes) | 0.580 |  |  | NA | 0.140 |  |  | NA |
| Tumor number (single vs. multiple) | **0.048** |  |  | NS | **0.029** |  |  | NS |
| Tumor size, cm (≤5 vs. >5) | **<0.001** | 3.162 | 2.182-4.582 | **<0.001** | **<0.001** | 2.374 | 1.744-3.232 | **<0.001** |
| Tumor encapsulation (complete vs. none) | 0.161 |  |  | NA | 0.098 |  |  | NA |
| Microvascular invasion (no vs. yes) | **<0.001** | 1.489 | 1.017 -2.179 | **0.041** | **<0.001** | 1.619 | 1.186-2.210 | **0.002** |
| Tumor differentiation (I-II vs. III-IV) | **0.029** |  |  | NS | **0.004** |  |  | NS |
| TNM stage (I vs. II III) | **<0.001** |  |  | NS | **0.029** |  |  | NS |
| 5-hmC (low vs. high) | **<0.001** | 0.583 | 0.399-0.853 | **0.005** | **<0.001** | 0.653 | 0.477-0.894 | **0.008** |
| IDH2 (low vs. high) | **0.001** | 0.595 | 0.414- 0.856 | **0.005** | **<0.001** | 0.702 | 0.522-0.946 | **0.02** |
| Combination of 5-hmC and IDH2 | **<0.001** |  |  | **0.007** | **<0.001** |  |  | **0.009** |
| I versus II | **0.001** | 1.814 | 1.034-3.184 | **0.038** | **0.008** | 1.750 | 1.129-2.711 | **0.012** |
| I versus III | **0.002** | 1.823 | 0.999-3.326 | **0.050** | **0.021** | 1.688 | 1.057-2.694 | **0.028** |
| I versus IV | **<0.001** | 2.678 | 1.538-4.663 | **<0.001** | **<0.001** | 2.154 | 1.378-3.366 | **0.001** |

Abbreviations: OS, overall survival; TTR time to recurrence; AFP, α-fetoprotein; γ-GT, γ-glutamyl transferase; TNM, tumor-nodes-metastasis; CI, confidential interval; NA, not adopted; NS, not significant. Boldface type indicates significant values.

†Cox proportional hazards regression.

I, 5-hmC ^High^/IDH2 ^High^; II, 5-hmC ^Low^/IDH2 ^High^; III, 5-hmC ^High^/IDH2 ^Low^; IV, 5-hmC ^Low^/IDH2 ^Low^. Boldface type indicates significant values.
